# Case report: Myocardial noncompaction causing massive cerebral infarction in 1 patient with eyelid edema as an early manifestation and literature review

**DOI:** 10.3389/fped.2023.1108570

**Published:** 2023-03-30

**Authors:** Pingping Ge, Yafei Zhu

**Affiliations:** Department of Pediatrics, Affiliated Hospital of Hangzhou Normal University, Hangzhou, China

**Keywords:** noncompaction of ventricular myocardium (NVM), children, massive cerebral infarction, clinical manifestations, prognosis

## Abstract

**Objective:**

To summarize and analyze the early clinical manifestations, risk factors, treatment and prognosis of myocardial noncompaction in children, and to provide scientific basis for early and effective intervention.

**Methods:**

Combined with a case of myocardial noncompaction with massive cerebral infarction in a child, the related research reports of myocardial noncompaction in children were analyzed retrospectively.

**Results:**

Myocardial noncompaction in children is cardiomyopathy caused by abnormal myocardial compaction during embryonic development. Feeding intolerance, dyspnea, chest tightness, fatigue, eyelid edema and other non-specific manifestations may occur in the early stage. It is easy to miss the diagnosis and misdiagnosis in clinical diagnosis and treatment, leading to intractable heart failure, nausea and arrhythmia, thromboembolism and even sudden death and other serious complications. Early diagnosis, symptomatic treatment, control of complications and regular follow-up can prevent the occurrence of serious complications and reduce mortality.

**Conclusion:**

There is no specific clinical manifestation in the early stage of myocardial noncompaction in children. If it is not detected early and treated symptomatically, the prognosis is poor and the mortality is high. Therefore, clinicians should fully improve the understanding of the early clinical manifestations of this disease, give early diagnosis and early intervention to children, reduce the occurrence of serious complications and improve the survival rate.

## Introduction

Noncompaction of ventricular myocardium (NVM) is a rare congenital inherited cardiomyopathy caused by endocardial dysplasia, and its occurrence and development have a complex genetic background. At present, the pathogenesis is still not very clear. The disease is characterized by a large number of abnormally thick trabeculae and deep recesses between trabeculae in the ventricle, so it is also called cavernous myocardium and honeycomb myocardium. Previous studies have suggested that the clinical manifestations are diverse and non-specific, mostly arrhythmia, cardiac insufficiency, thrombosis and sudden death as the main manifestations, and early diagnosis and timely treatment of this disease are essential. Clinical reports of NVM with cerebral infarction in children are extremely rare.

**Figure 1:**
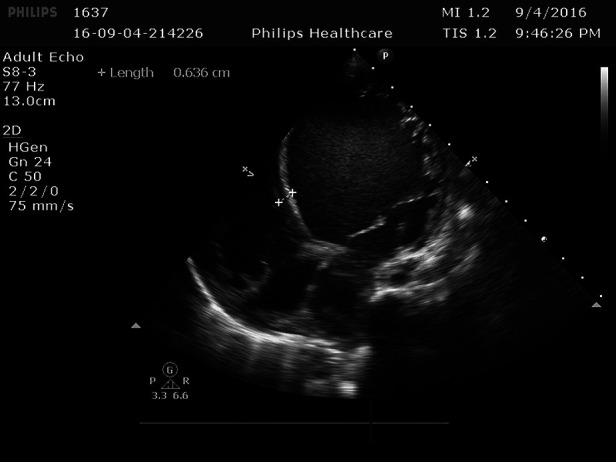
Ventricular septal thickness0.636 cm.

**Figure 2:**
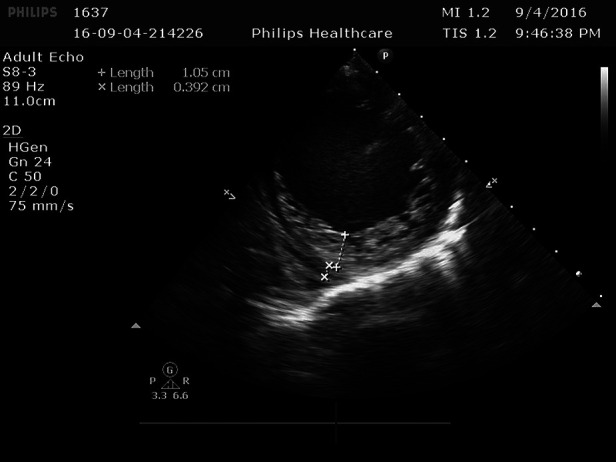
In the left ventricle, trabeculae increased significantly and protruding into the ventricle cavity, and deep recess could be seen. The thickness of the non-dense myocardium was 10.5 mm, and the thickness of the dense myocardium was 3.9 mm, and the ratio of the two was >2.

**Figure 3:**
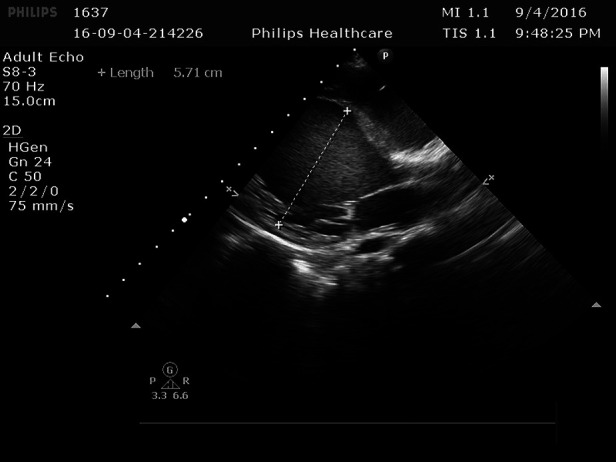
The left ventricle was significantly enlarged 5.71 cm.

**Figure 4:**
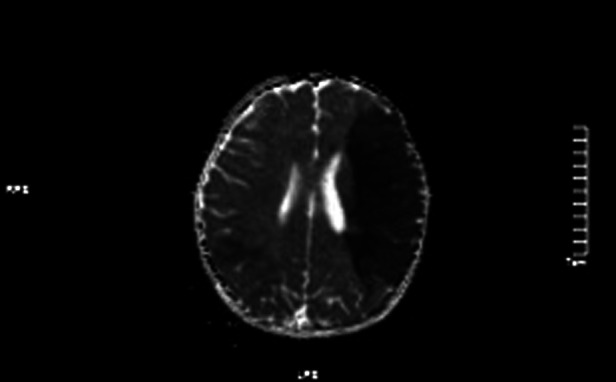
ADC values decreased in the left frontotemporal lobe and the right temporo-occipital lobe.

**Figure 5:**
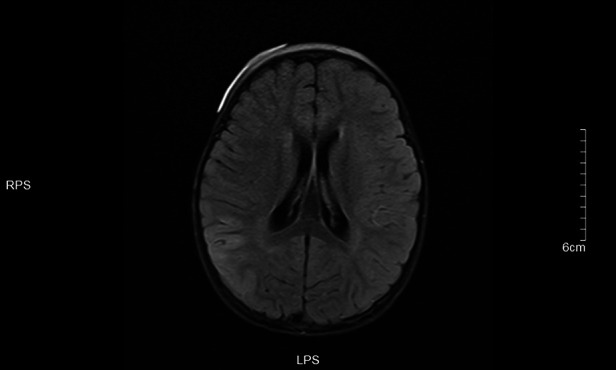
Left frontotemporal lobe and right temporo-occipital lobe T1 in Dark-fluid sequence slightly swollen left frontotemporal lobe and right temporo-occipital lobe gyri with increased signals.

**Figure 6:**
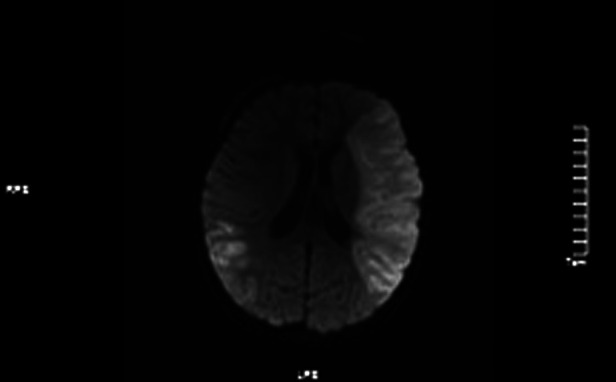
DWI images showed high signals in the left frontotem poral lobe and the right temporal.

**Figure 7:**
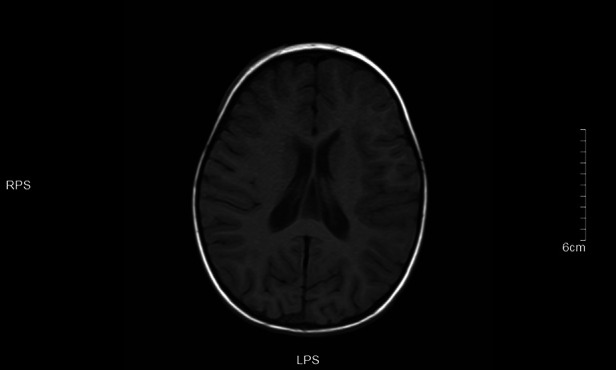
T1 left frontotemporal lobe and right temporal occipital lobe local gyri Slightly swollen.

**Figure 8:**
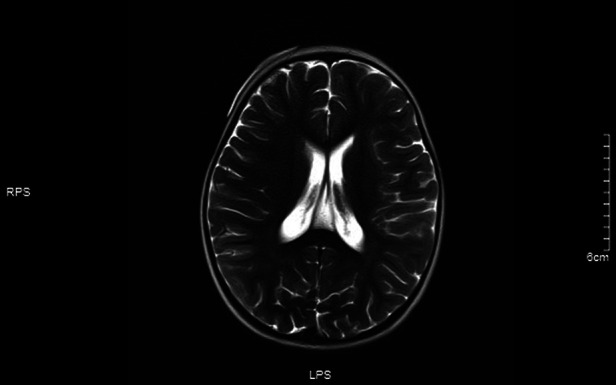
No significant abnormality was observed in T2 sequence signal.

## Materials and methods

A 21-month-old female child was admitted to our hospital with immobility of the right limb associated with inability to speak for 1 h. One hour ago, the child suddenly developed right limb immobility, inability to stand, with inability to speak, and deviation of the mouth to the left, and recurrent head and right lower limb twitching after admission. He had no fever, no headache or vomiting, and denied any history of cranial trauma. Past medical history: In July 2021, the child visited another hospital due to pneumonia, and chest x-ray showed enlarged heart shadow. Since October 2021, he had bilateral eyelid edema, which was evident in the morning. Eyelid edema had not resolved for several months. Family history: denied any family history of hereditary diseases. Birth history: She was born at term by spontaneous delivery and denied any history of asphyxia rescue. Growth and development history: The growth and development of the child were the same as those of the child of the same age. Admission examination showed T 36.2°C, HR 122 beats per minute, RR 48 breaths per minute, BP 99/64 mmHg, clear consciousness, soft spirit, aphasia, bilateral eyelid edema, bilateral pupils of equal size and round, sensitive light reflexes, shallow nasolabial folds on the right side, regular rhythm, moderate heart sounds, no murmur heard in the precordium, and no special pulmonary or abdominal examination. Muscle strength was grade V in the left upper and lower limbs and grade II in the right upper and lower limbs. Auxiliary examination: blood coagulation function was basically normal; on January 7, 2022, biochemistry (emergency): total bilirubin: 3.9 μmol/L, total protein: 35.6 g/L, albumin: 20.8 g/L, alanine aminotransferase: 20 U/L, aspartate aminotransferase: 49 U/L, creatine kinase: 132 U/L, creatine kinase isoenzyme (mass method): 6.95 ng/ml, urea: 3.22 mmol/L, creatinine: 22.1 μmol/L; blood routine + SCRP showed no significant abnormality;

Head CT showed that the sulcal fissure in the left temporal region seemed to be slightly shallower, and MR examination was performed when clinically necessary.

MRI: (1). acute massive cerebral infarction in the left frontal lobe and bitemporoparietal occipital lobe.

Cardiac ultrasound: (1). interventricular septum thickness was fair. The motion amplitude of left ventricular wall was generally significantly reduced. Trabeculae were significantly increased and protruded into the ventricular cavity at the apex, lateral wall and inferoposterior wall of left ventricle, and deep lacunae were observed. The thickness of non-compacted myocardium was 10.5 mm and the thickness of compacted myocardium was about 3.9 mm. The ratio of the two was >2. CDFI showed that the blood flow signal communicating with the cardiac chamber in the recess. Fluid dark areas were observed in the pericardial cavity, measuring 7 mm in the dark area of the posterior wall of the left ventricle, about 10 mm in the dark area at the top of the right atrium, and about 8 mm in the dark area of the lateral wall of the right ventricle. Cardiac ejection fraction was 28%.

After admission, the patient was given dehydration, lowering intracranial pressure, static shock, trophic nerve and other symptomatic and supportive treatment. Thrombolytic therapy and subcutaneous injection of heparin sodium for antiplatelet therapy were immediately given after the presence of massive cerebral infarction. After treatment, the child had no recurrent convulsions. After treatment for about 12 days, the child's right upper and lower limb muscle strength improved to grade III, and was discharged after long-term oral aspirin antiplatelet therapy. At present, the child patient had no recurrence of cerebral infarction, but the muscle strength of right limb was still grade III, accompanied by movement limitation and slurred speech.

## Discuss

Myocardial noncompaction (NVM) is cardiomyopathy caused by abnormal cessation of myocardial compaction during embryonic development, and in 2006 the American Heart Association classified NVM as an independent cardiomyopathy. It accounts for 5% of cardiomyopathy in children (1). The pathogenesis is not yet fully understood, 17% to 50% of patients have a family history of cardiomyopathy (2), and their mode of inheritance is diverse, with autosomal dominant and X linked inheritance being the most common. The clinical manifestations are not obviously specific, and most cases may even have no clinical symptoms in the early stage, so they are easily misdiagnosed and missed in clinical work, and their long-term prognosis is poor, and many serious complications may occur. The main causes of death are malignant arrhythmia, refractory heart failure and thromboembolic events (3, 4). At present, there is still no specific treatment for NVM, so identifying its early clinical manifestations, early diagnosis, symptomatic treatment, and prevention of complications are the key to improve the survival rate of children.

The clinical manifestations of NVM are diverse and non-specific, and the clinical manifestations in the early stage of the disease are not easily detected or do not attract the attention of parents and doctors, and many children present with this disease after the corresponding manifestations of significant arrhythmia or severe cardiac insufficiency. Some of them were diagnosed by pedigree screening due to the presence of cases of noncompaction of the myocardium in the family.

The early symptoms of the child in this case were not described by the parents and their first physician until the child was found to have enlarged heart shadow when the chest radiography examination was perfected due to pneumonia half a year before the onset of the disease, but the parents did not pay attention to it and did not perfect the cardiac ultrasonography. Three months later, the child developed bilateral eyelid edema, and the child developed cerebral infarction 3 months after the onset of the symptoms. Ultrasonography showed that the apical, lateral and posterior walls of the left ventricle were involved, and the extent of involvement was large, so the child's condition progressed rapidly, and massive cerebral infarction was caused by thrombosis 3 months after the onset of clinical symptoms. Reviewing the course of the disease, bilateral eyelid edema is a manifestation of heart failure, when cardiac systolic and diastolic function are impaired, which leads to increased circulating venous pressure and tissue hydrostatic pressure, causing organ and tissue edema leading to bilateral eyelid edema. At this time, the child may also have shortness of breath, dyspnea, movement limitation, limb edema and other manifestations of heart failure, but the parents of the child did not notice whether the child had corresponding manifestations in daily life. In the process of two visits due to eyelid edema, the parents of the child did not provide the information that the child had enlarged heart shadow found by previous examination. At the same time, the doctor did not know enough about the possibility of eyelid edema as a heart problem, resulting in failure to timely improve the relevant examinations such as cardiac ultrasound and cardiac function, so the disease could not be detected early and timely symptomatic treatment was given, eventually leading to massive cerebral infarction in the child.

In this case, there were multiple opportunities for early detection and diagnosis when physical examination revealed enlarged heart shadows and eyelid edema, but they were missed because clinicians did not fully recognize the disease. Reviewing the literature, we found that cardiac dysfunction is the most common clinical manifestation in children with NVM, and its incidence can be as high as 87% in children (5). Ichida (6) et al. conducted a 17-year survey of 27 children with NVM found by physical examination and found that 14.8% had symptoms in the early stage, while 59.3% had varying degrees of cardiac dysfunction after 17 years. A Japanese long-term prognosis follow-up survey of 205 children with myocardial noncompaction found that 60% of children under 1 year of age had developed significant cardiac dysfunction at the first diagnosis (3). Cardiac insufficiency is mainly caused by chronic myocardial ischemia due to thick trabeculae and spaces, and subendocardial myocardial fibrosis, which affects ventricular contraction and relaxation. The time and severity of symptoms are related to the extent of myocardial involvement, and the onset of lesions localized in the apex of the left ventricle is relatively late, and if most of the left ventricle is involved, the symptoms appear early and significantly (7). The early manifestations of cardiac insufficiency vary in children of different ages, and infants mostly present with difficulty feeding, reduced milk volume, shortness of breath, irritability, and oliguria; while older children mostly present with chronic cardiac insufficiency such as chest pain, fatigue, cough, chest tightness, palpitations, syncope, and dyspnea (8, 9). Therefore, in clinical work, when we encounter children without obvious underlying diseases who present with manifestations of cardiac insufficiency, we should fully consider the possibility of this disease, and improve cardiac ultrasonography, diagnose as early as possible, and treat in time to prevent the occurrence of serious complications.

Thrombosis is the main cause of serious adverse prognosis such as pulmonary embolism, cerebral infarction and peripheral embolism in children with NVM, and its incidence varies from 0%–38% (6, 10–14). Decreased left ventricular systolic function has been found to be the most important cause of thrombosis and thrombus formation tends to occur when systolic ejection fraction is <40%. Thromboembolic events occur in only 3% of children after thrombosis (11, 12), and even most studies have reported no thromboembolic events (10, 15), while NVM complicated by cerebral infarction in children is extremely rare and has not been reported in China. In this case, the child presented with typical manifestations of cerebral infarction such as limb immobility and speech inability, and cardiac ultrasonography after presentation revealed findings consistent with myocardial noncompaction, and the child's cardiac ejection fraction was only 28%, significantly decreased, and there were manifestations of heart failure, so it was considered that the child had thrombosis due to myocardial noncompaction and decreased cardiac function, resulting in the occurrence of cerebral infarction. There are not many clinical cases of NVM complicated with cerebral infarction, and its main clinical manifestations are tantamount to cerebral infarction induced by other causes, but seriously affect the quality of life and prognosis of children with NVM. Brazilian guideline (16) recommends long-term anticoagulant therapy in patients with systolic ejection fraction < 40%, previous history of cerebral embolism or atrial fibrillation, heparin sodium and warfarin (6, 13) are the drugs of choice, and there are no relevant reports of bleeding side effects (5, 17–20). Aspirin is effective in preventing thrombosis with relatively low bleeding adverse effects and can be used in all children with NVM (13, 21).

In addition to the above clinical manifestations, arrhythmia is a common clinical manifestation in children with NVM, especially in older children. Tachyarrhythmia is more common in clinical practice, mainly Wolff-Parkinson-White syndrome, atrioventricular block and ventricular tachycardia. Among them, second-degree atrioventricular block is only seen in children, and no adult morbidity has been reported (3, 22). Ozgur et al. (5) found that growth retardation was the most common extracardiac manifestation in children with this disease and was mostly associated with cardiac dysfunction. In addition, it has also been reported that NVM is closely related to neuromuscular diseases (neuromusculardisorder, NMD), a study by Stöllberge et al. (23) found that half of NVM patients have unexplained NMD, while about one-third of patients have specific NMD, which also reminds us that there may be some common pathogenesis between NVM and NMD. At present, there is no report of NVM with NMD in China, but we should be vigilant, and corresponding examinations should be perfected for NVM patients to determine whether they have NMD in clinical work.

## Summary

NVM in children is an independent congenital cardiomyopathy. At present, there are not many relevant clinical reports and studies, and clinicians generally lack of understanding of this disease, so it is easy to miss the diagnosis and misdiagnosis. The prognosis of NVM varies greatly, and those without clinical manifestations can survive for a long time; those with young age of onset and severe clinical symptoms have a poor long-term prognosis and can experience serious complications such as refractory heart failure, thromboembolism, nausea and arrhythmia and sudden death, with a high mortality rate. Although there may be no specific clinical symptoms in the early stage of the disease, most children present with non-specific manifestations such as feeding difficulties, fatigue, dyspnea, cough, and chest tightness, and a few children have cardiac insufficiency manifestations such as hepatomegaly, lower limb and eyelid edema at presentation. Therefore, in clinical work, if children without obvious underlying diseases present with cardiac insufficiency as the main manifestation, clinicians should be vigilant and must improve the examination to determine whether it is NVM. Early diagnosis, symptomatic treatment and long-term follow-up can effectively improve the prognosis of children, improve life treatment and reduce their mortality.

## Data Availability

The original contributions presented in the study are included in the article/Supplementary Material, further inquiries can be directed to the corresponding author/s.
